# Photodynamic Therapy of Cancers With Internal Light Sources: Chemiluminescence, Bioluminescence, and Cerenkov Radiation

**DOI:** 10.3389/fchem.2020.00770

**Published:** 2020-09-04

**Authors:** Yintang Zhang, Yuanqiang Hao, Shu Chen, Maotian Xu

**Affiliations:** ^1^Henan Key Laboratory of Biomolecular Recognition and Sensing, College of Chemistry and Chemical Engineering, Henan Joint International Research Laboratory of Chemo/Biosensing and Early Diagnosis of Major Diseases, Shangqiu Normal University, Shangqiu, China; ^2^Key Laboratory of Theoretical Organic Chemistry and Function Molecule of Ministry of Education, School of Chemistry and Chemical Engineering, Hunan University of Science and Technology, Xiangtan, China

**Keywords:** photodynamic therapy, cancers, internal light sources, chemiluminescence, bioluminescence, Cerenkov radiation

## Abstract

Photodynamic therapy (PDT) is a promising and minimally invasive modality for the treatment of cancers. The use of a self-illuminating system as a light source provides an intriguing solution to the light penetration issues of conventional PDT, which have gained considerable research interest in the past few years. This mini review aimed to present an overview of self-illuminating PDT systems by using internal light sources (chemiluminescence, bioluminescence, and Cerenkov radiation) and to give a brief discussion on the current challenges and future perspectives.

## Introduction

Photodynamic therapy (PDT) is a promising non-invasive medical technique and has been clinically approved for treating various diseases, including bacterial and fungal infections, skin diseases, as well as several types of cancer (van Straten et al., 2017). In the past decades, advances in nanotechnology and materials science as well as the improvements of photosensitizers (PS) have promoted the rapid development of PDT (Chen et al., 2017; Glass et al., 2018; Ouyang et al., 2018a,b; Fan et al., 2019; Yue et al., 2019; Zeng et al., 2019). However, the conventional external light irradiations in PDT often suffer from rapid attenuation through the tissue, which limited the clinical use of PDT to some superficial or endoscope-accessible lesions. To overcome this barrier, different excitation sources, such as near-infrared (NIR) light and X-ray radiation, have been applied in combination with a number of judiciously designed photosensitizers, such as two-photon (Shen et al., 2016) or NIR light (Lan et al., 2019) excitation photosensitizers and upconversion photosensitizers (Liu et al., 2019). Nevertheless, it is still challenging to develop NIR photosensitizers with high absorption efficiency, and NIR light also has penetration limitation (i.e., 980-nm light is restricted to 1.5 cm). X-ray radiation, photon energy in the range of 0.1–100 keV, can overcome the limitation of the light penetration depth in the human body. But X-rays can ionize atoms and disrupt chemical bonds of normal biomolecules. Implanting fiber-optic light sources could be a viable approach to treat deep tissue, but it requires invasive procedures and cannot deal with tumor metastasis.

**Figure 1:**
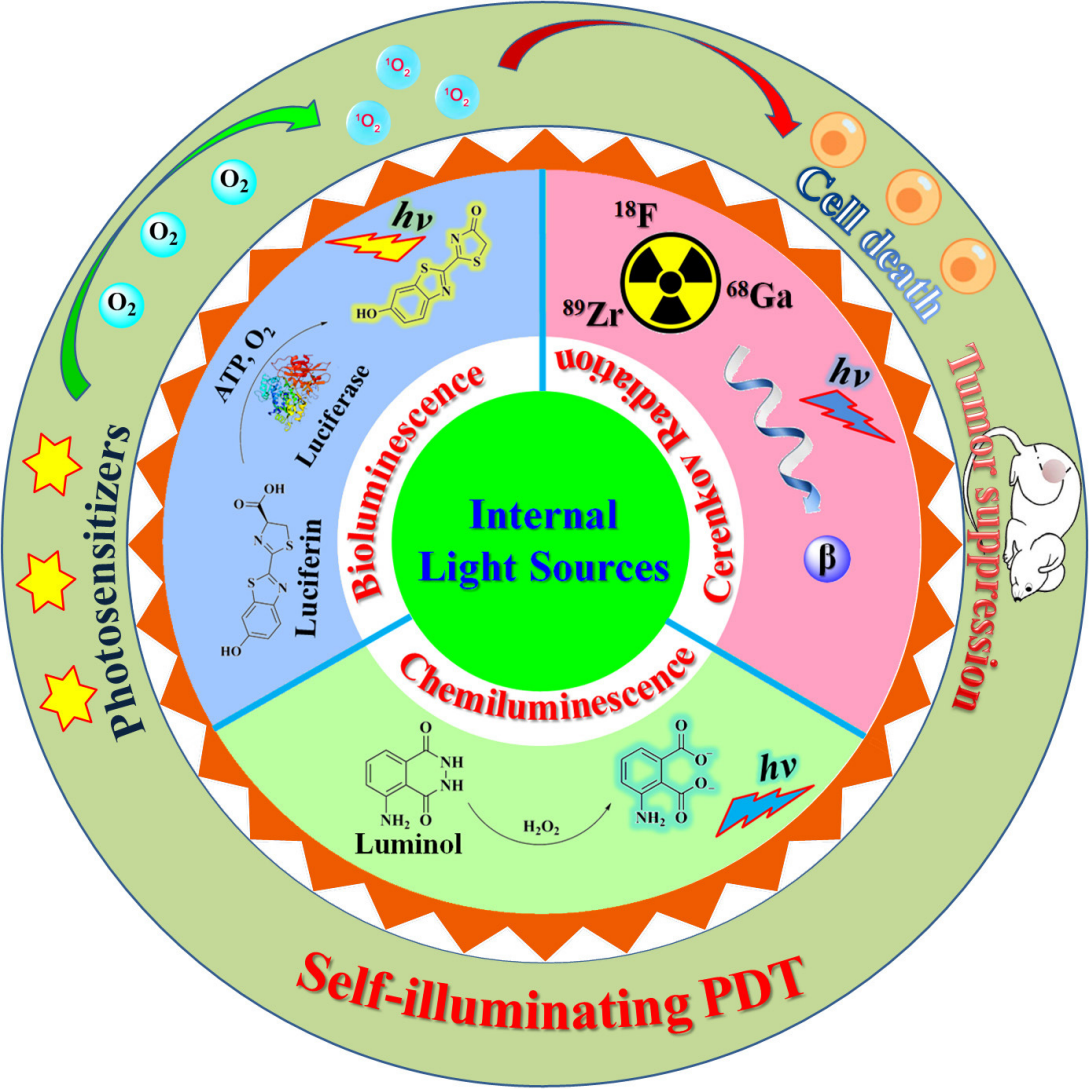
Illustration of a self-illuminating photodynamic therapy (PDT) system based on internal light sources, including chemiluminescence, bioluminescence, and Cerenkov radiation.

Internal light sources have emerged as an attractive alternative to outer light sources in a conventional PDT system for addressing the issue of light penetration (Magalhães et al., 2016; Ferreira et al., 2019). Some self-illuminating systems, including chemiluminescence (CL), bioluminescence (BL), and Cerenkov radiation (CR), are promising candidates as internal light sources for PDT as these self-illuminators are small in size (ranging from atomic/molecular to nanometer scale) and thus can be delivered to any pathological tissues. The use of self-illuminating systems as light sources in PDT has attracted increasing research interest in the past few years (Fan et al., 2016; Magalhães et al., 2016; Jiang et al., 2020). In this mini review, we summarize recent progress in the development of self-illuminating PDT systems, including chemiluminescence-, bioluminescence-, and Cerenkov radiation-mediated photodynamic therapy (Figure 1). The design strategies and chemical structures of these reported PDT systems will be emphasized. Current limitations and future directions will also be discussed.

## Chemiluminescence-Mediated Photodynamic Therapy

CL is a kind of self-luminescence phenomenon that results from some special types of chemical reactions. Up to now, chemiluminescence reactions have found a wide variety of applications (Iranifam, 2014; Shah et al., 2020) such as chemosensing, bioimaging, and therapy. The most commonly used chemiluminescence systems are luminol–H_2_O_2_ and oxalate ester–H_2_O_2_.

The first report of using luminol chemiluminescence as an excitation light source for PDT application was demonstrated by Laptev et al. (2006). A transferrin–hematoporphyrin (Tf–Hp) conjugate was used as the cancer cell targetable photosensitizer. The absorption spectra of Tf–Hp (one absorption peak at 412 nm) overlapped well with the CL emission band of luminol (350–550 nm). The addition of luminol can induce a noticeable intracellular CL over a 50-min period. The combination of luminol (10 μM) and the Tf–Hp conjugate (3 μM) resulted in 95% cytotoxicity against the erythroleukemic cell lines.

Yuan et al. (2012) developed a new chemiluminescence-mediated PDT by using a cationic oligo(*p*-phenylene vinylene) (OPV) as the photosensitizer. The cationic OPV could not only interact with the dianionic intermediates of luminol CL reaction to facilitate the occurrence of chemiluminescence resonance energy transfer (CRET) to excite OPV but could also bind to the negatively charged pathogen (cancer cells and fungi). The luminol chemiluminescence system with OPV displayed prominent cytotoxicity toward HeLa cells with a viability of <10%. Studies in tumor-bearing nude mice indicated that this self-illuminated PDT system could significantly inhibit the tumor growth *in vivo* without exerting apparent toxicity to the normal tissues. Based on the cationic OPV photosensitizer, the same research group proposed a novel electrochemiluminescence-mediated PDT system (Liu et al., 2018). Recently, Jiang's group reported a conjugated polymer nanoparticle-based multifunctional nanoplatform for oxygen-supplying and self-illuminating phototherapy (Jiang et al., 2019). The nanoplatform Hb–NPs was obtained by covalently coupling hemoglobin (Hb) to the polymer nanoparticle consisting of poly(styrene-*co*-maleic anhydride) (PSMA) and poly[2-methoxy-5-(2-ethylhexyloxy)-1,4-phenylenevinylene] (MEH–PPV). The polymer-conjugated Hb can simultaneously function as the oxygen carrier, the catalyst for the CL reaction of luminol with H_2_O_2_, as well as the PDT photosensitizer for generating reactive oxygen species (ROS). Thus, the presented system does not require an external light source and can overcome the problem of insufficient oxygen under hypoxia. In addition, the system can also be used to control the release of an anticancer prodrug, thus providing simultaneous phototherapy and chemotherapy.

The combination of different forms of photosensitizers with luminol CL has afforded a variety of other internal self-illuminating PDT systems, including tetra(hydroxyphenyl)chlorin (THPC)-encapsulated FH-Pdots [folic acid and horseradish peroxidase (HRP)-bifunctionalized polymer dots] (Zhang et al., 2014), 5-aminolevulinic acid (Chen et al., 2012), porphyrinic metal–organic frameworks (C_O_TCPP MOFs) (Fang et al., 2019), chlorin e6-conjugated yellow-emissive carbon dots (y-CDs-Ce6) (Yang et al., 2020), and poly(lactic-*co*-glycolic acid) (PLGA)/luminol/HRP/DSPE-mPEG2000 co-precipitated nanoparticles (Lu et al., 2020). Covalent attachment of luminol to the photosensitizer into a single molecule is an additional strategy for constructing a CL-PDT system through the direct energy transfer from luminol to the photosensitizer. Yesilgul et al. (2017) designed a modular unimolecular erythrosine–luminol which can produce singlet oxygen in the presence of Cu^2+^ and hydrogen peroxide. Xu et al. (2019) synthesized a Ce6–luminol–PEG (CLP) conjugate which can self-assemble into core–shell nanoparticles. The nanoparticle can be exploited for inflammation imaging as well as for specifically killing cancer cells. In addition to the commonly used chemiluminophore of luminol, peroxyoxalate chemiluminescent (PO-CL) reaction also has the potential to supply internal light source for PDT. Romanyuk et al. (2017) developed a self-luminescing PDT strategy by using polymeric oxalate (POX) as the substrate of the PO-CL reaction and tetramethyl hematoporhyrin (TMHP) as both a photosensitizer and an activator for the PO-CL reaction. POX and TMHP were dispersed in dimethyl phthalate (DMP) droplets and stabilized with a surfactant of Pluronic L64. The L64/DMP/POX/TMHP dispersions can effectively produce ${\text{O}}_{2}^{1}$ and exert significant cytotoxicity under oxidative stress condition. Mao et al. (2017) designed a novel nanoplatform [C-TBD nanoparticles (NPs)] for image-guided PDT by co-encapsulating bis[2,4,5-trichloro-6-(pentyloxycarbonyl)phenyl]oxalate (CPPO) and the photosensitizer TBD into an amphiphilic copolymer Pluronic F-127. The astutely designed photosensitizer TBD displayed a bright aggregation-induced NIR emission and an efficient singlet oxygen generation. C-TBD NPs could precisely monitor the tumor site via chemiluminescence imaging and efficiently inhibit tumor growth through PO-CL exciting TBD to produce ${\text{O}}_{2}^{1}$ in the presence of H_2_O_2_, thus realizing simultaneous tumor diagnosis and treatment. Coelenterazine and its analogs can also act as a self-activating agent for PDT (Pinto da Silva et al., 2019; Sun et al., 2019). In the presence of a superoxide anion (overexpressed in tumor cells), coelenterazine can be oxidized into a dioxetanone intermediate, which then decomposing into CO_2_ and the excited coelenteramide. The product will further undergo intersystem crossing to the triplet state, thus inducing the generation of ${\text{O}}_{2}^{1}$. This process can be enhanced by the introduction of a bromine heteroatom due to the heavy atom effect (Pinto da Silva et al., 2019).

## Bioluminescence-Mediated Photodynamic Therapy

Bioluminescence can be considered as a special type of CL which involves the enzymatic oxidation of a small molecule substrate, such as the firefly luciferase–luciferin, and Renilla luciferase–coelenterazine systems (Magalhães et al., 2016; Hananya and Shabat, 2017). Theodossiou et al. firstly evaluated the potential of intracellular bioluminescence as a light source for PDT (Theodossiou et al., 2003). The classical firefly luciferin–luciferase system was used to excite the photosensitizer, Rose Bengal (RB), which has a high singlet oxygen quantum yield and a compatible absorption profile with the emission of oxyluciferin. Treating the luciferase-transfected NIH 3T3 cells with RB and D-luciferin led to a high rate of apoptosis (89%). This preliminary *in vitro* study demonstrated the possibility of a bioluminescence-mediated PDT to be a plausible treatment modality. However, the following work by Schipper et al. (2006) revealed that luciferase-expressing cells incubated with photosensitizer and D-luciferin did not show a significant difference in survival compared with the control groups. The authors demonstrated that this intracellular bioluminescence system cannot emit enough photons (<1.03 × 10^−4^ mJ cm^−2^ for 24 h treatment) to mediate photosensitizers to generate efficacious photodynamic toxicity.

In the above cases, the bioluminescent enzyme appeared in cells through gene expression, a technology that has not been applied in actual clinical treatment, so this bioluminescent system has difficulty in becoming an alternative excitation for PDT in the near future. Hsu et al. (2013) developed a new coelenterazine bioluminescence-mediated PDT system based on the bioluminescent QD-Rluc8 conjugate which was obtained by immobilizing Renilla luciferase onto quantum dot 655. In the presence of coelenterazine, QD-Rluc8 can display the distinct fluorescence emission of quantum dots (QDs) via bioluminescent resonance energy transfer (BRET) from the bioluminescent substrate to the QDs. Both *in vitro* and *in vivo* assays demonstrated that the bioluminescent QD-Rluc8 can stimulate the micelle-loaded photosensitizer meta-tetra(hydroxyphenyl)chlorin (m-THPC, Foscan) to produce ROS and thus result in cell death and inhibition of tumor growth. As a potential alternative excitation source for PDT, QD-Rluc8 displayed several advantages, including a tunable emission wavelength and a relatively high irradiation dose (0.6–0.8 J cm^−2^). Subsequently, Kim et al. (2015) evaluated whether BL can induce efficient PDT for tumors, especially in deep tissue. A similar BRET Luc-QD luminophore was employed as the BL source, and Ce6 was used as the photosensitizer. Confocal imaging showed that Luc-QD conjugates were mainly distributed on the cell surface instead of in the cytoplasm. Calculating from the emission spectra, the BRET efficiency of the Luc-QD system reached 60–65%. Luc-QD can excite the nearby photosensitizer, leading to an appreciable amount of activated Ce6 (3 × 10^8^/min), which is higher than that (4 × 10^7^/min) resulting from laser illumination (2.2 mW/cm^2^). Cell imaging and cytotoxicity tests revealed that this BL-PDT system can generate significant intracellular ROS, thus resulting in membrane damage and cell death. Intravenous injection of Ce6, Luc-QD, and CTZ to tumor-implant mice can almost completely inhibit tumor growth. Furthermore, this BL-PDT can also suppress distant organ metastasis. Recently, Yang et al. (2018) reported a new BL-PDT system based on the polymer nanoparticle which was assembled from poly(lactic acid) and poly(lactic-*co*-glycolic acid) and was loaded with Rose Bengal and luciferase. *In vitro* photodynamic studies showed that this BL-PDT treatment can lead to significant toxicity toward cancer cells. *In vivo*, BL-PDT displayed that the growth of subcutaneous tumors can be remarkably inhibited, while normal organs, including the heart, liver, spleen, lung, and kidney, remained undamaged.

## Cerenkov Radiation-Induced Photodynamic Therapy

Cerenkov radiation (CR) is a luminescence phenomenon occurring from the interaction between high-speed charged particles (faster than the phase velocity of light) with the surrounding medium, which was theoretically predicted by Heaviside in 1888 and experimentally verified by Cerenkov in 1933 (Shaffer et al., 2017). In biomedical applications, the commonly used sources for producing Cerenkov radiation are radionuclides which can emit β particles (positrons or electrons) (Gill et al., 2015), such as ^18^F, ^64^Cu, ^68^Ga, ^89^Zr, ^90^Y, ^124^I, and ^198^Au. The number of Cerenkov photons within a certain wavelength range is positively correlated to the kinetic energy (or velocity) of the β particle. Radionuclides emitting β particles with relatively higher energy, such as ^68^Ga and ^90^Y, would produce CR with higher intensity (Klein et al., 2019). In recent years, the Cerenkov phenomenon for biomedical applications (i.e., imaging and therapy) has gradually garnered significant attention, especially combining the rapid advancements in nanosciences and nanotechnologies (Shaffer et al., 2017; Cline et al., 2019; Ferreira et al., 2019).

In 2015, Kotagiri and coworkers reported a proof-of-concept study using CR from a common diagnostic clinical radiotracer, 2′-deoxy-2′-[^18^F]fluoro-d-glucose ([^18^F]-FDG), as the light source for PDT, denoted as CR-induced therapy (CRIT) (Kotagiri et al., 2015). By incorporating both apotransferrin (Tf) and titanocene (Tc) into TiO_2_ nanoparticles, the authors designed and prepared an effective nanophotosensitizer (TiO_2_-Tf–Tc) which can function with low-intense radiation. TiO_2_ nanoparticles have the capability to effectively utilize CR, which locates predominantly within the ultraviolet to the blue spectral region (characteristic 1/λ^2^ spectrum), to generate hydroxyl and superoxide radicals. Tf can not only act as a stabilizer and dispersant for TiO_2_ but also as a tumor-targeting agent. Tc can serve as a complementary photosensitizer for generating cyclopentadienyl and titanium-centered radicals. *In vitro* cellular experiments have shown that TiO_2_-Tf can be endocytosed by tumor cells and, together with [^18^F]-FDG, can lead to an appreciable decrease in cell viability. *In vivo* studies have demonstrated that the administrated TiO_2_-Tf–Tc prefer to accumulate in the tumor tissue and that the co-administration of TiO_2_-Tf–Tc and radionuclides efficiently inhibited the tumor growth and increased the median survival. These results demonstrated the possibility of utilizing CR as the internal exciting light for PDT photosensitizers, and this pioneering work opened a new avenue for the application of the Cerenkov effect in PDT, especially in a depth- and oxygen-independent manner. Following this study, Duan et al. (2018) reported another CRIT system by using ^68^Ga-labeled bovine serum albumin (^68^Ga-BSA) as a more efficient Cherenkov radiation emitter and dextran-modified TiO_2_ nanoparticles (D-TiO_2_ NPs) as the nanophotosensitizer. For comparison, [^18^F]-FDG was also used throughout this study. Positron emission tomography (PET) images of tumor-bearing mice intratumorally injected with radionuclides revealed that both ^68^Ga-BSA and [^18^F]-FDG can predominantly stay in the tumor in similar amounts, whereas much more CR photon accumulation appeared in mouse loaded with ^68^Ga-BSA. As a result, ^68^Ga-BSA with D-TiO_2_ exhibited a significantly enhanced PDT efficacy. Injection of ^68^Ga-BSA and D-TiO_2_ NPs into the tumor mass caused complete suppression of the tumor growth and a significant increase in median survival, while no obvious efficacy was observed for the other group, even with the combination of [^18^F]-FDG and D-TiO_2_ NPs.

In the above two studies, the Cerenkov source (radiotracer) and the PDT photosensitizer were used individually, as two detached elements. After administration, the radiotracer and photosensitizer would randomly distribute in the target location and within a relatively large distance. Thus, a large portion of the Cerenkov photons from the radiotracer may have probably been absorbed or scattered by the media instead of exciting the photosensitizer. To circumvent this problem, Kamkaew et al. (2016) designed an integrated CRIT system, [^89^Zr]HMSN-Ce6, by loading the Cerenkov source (^89^Zr) and photosensitizer (Ce6) into hollow mesoporous silica nanoparticles (HMSNs). *In vitro* imaging displayed that [^89^Zr]HMSN-Ce6 can emit intense fluorescence (690–710 nm) in solution without external excitation light, which clearly confirmed the occurrence of energy transfer between ^89^Zr and Ce6. *In vivo* CR-induced PDT studies further confirmed the efficacy of [^89^Zr]HMSN-Ce6 to completely inhibit tumor growth. Subsequently, the same research group proposed a magnetic targetable CRIT system (Ni et al., 2018), ^89^Zr-MNP/TCPP, which was prepared by labeling PEGylated magnetic nanoparticles (MNP) with ^89^Zr via Lewis acid–base interaction and then conjugating with the photosensitizer, meso-tetrakis(4-carboxyphenyl)porphyrin (TCPP), via amide coupling. Various imaging analyses (including PET, fluorescence, CL, and CRET) confirmed that the magnetic nanoparticles can be accumulated in tumor regions under an external magnetic field. The ^89^Zr-MNP/TCPP ternary composite has shown efficient ^1^O_2_ generation efficiency as well as a high magnetic-guided therapeutic efficacy. Recently, Yu's group proposed a novel “missile detonation” strategy for efficient CR-induced theranostics by the successive administration of a high dose of porphyrin–PEG nanocomplex (PPN) and a low dose of ^89^Zr-labeled PPN (^89^Zr-Df-PPN) (Yu et al., 2019). The PPN and ^89^Zr-Df-PPN acted as the CR energy receiver/missile and ^89^Zr-Df-PPN the CR energy donor/detonator, respectively. These therapeutic agents can be monitored via multimodal imaging, including fluorescence imaging, CRET imaging, and PET. This CRIT system could significantly inhibit tumor growth *in vivo* and cause substantial fragmentation of vascular in the tumor.

## Conclusions and Perspectives

In this review, we have summarized recent advances in the development of self-illuminating PDT systems. Internal light sources, including chemiluminescence, bioluminescence, and Cerenkov radiation, have been proven capable of exciting certain photosensitizers to produce ROS and generate efficacious photodynamic toxicity. The use of these internal light sources has great potential for depth-independent PDT, which would expand the scope of PDT in cancer treatment.

Despite the impressive progress outlined above, the current self-illuminating PDT systems still face several challenges, including a relatively low photon flux, the requirement of multicomponent reactions for CL and BL, limited wavelength range of the excitation light, etc. To overcome these shortcomings, several future directions for the design of self-illuminating PDT systems are proposed as follows:

(1) Nanocarrier technologies can be employed for increasing the amount of administrated self-illuminators. If the reactants for producing internal light, such as luminol, luciferase–luciferin pairs, and CR radiotracer, were loaded and delivered to the tumor tissues by proper nanocarriers, they would provide a higher local photon flux and, thus, more efficient PDT. The use of multifunctional nanoplatforms for a targeted delivery and controlled release of these illuminators is also recommended.

(2) Nanophotosensitizers are more suitable for self-illuminating PDT systems as they (metal oxide nanocrystals, QDs, polymer dots, etc.) have higher cross-sections for absorbing light compared with single small-molecule photosensitizers. Moreover, nanophotosensitizers, such as QDs, can be tuned to absorb light with wavelengths ranging from near-UV through visible to near-IR.

(3) The currently used internal light sources can be further expanded. Compared with the conventional CL systems of luminol–H_2_O_2_ and oxalate ester–H_2_O_2_, the newly developed triggerable dioxetanes are more attractive chemiluminescent probes (Hananya and Shabat, 2017) as they have tunable structures and emission features, high CL quantum yields, without the requirement of an enzyme/catalase and H_2_O_2_. Thus, triggerable dioxetanes hold great potential as internal light sources for PDT. The use of a structure-modified luciferin as the substrate is a desirable approach for tuning the emission wavelength of BL, which would expand the application of BL in PDT.

(4) The combined theranostic strategy is also an exciting trend for improving the efficacy of self-illuminating PDT as the integration of two or more treatments can generate synergistic effects for antitumor therapy. Thus, a promising future direction is to combine self-illuminating PDT with various other treatments, such as immunotherapy, chemotherapy, and photothermal therapy.

## Author Contributions

YH and YZ organized and wrote the manuscript. SC and MX discussed the results. All authors approved this manuscript.

## Conflict of Interest

The authors declare that the research was conducted in the absence of any commercial or financial relationships that could be construed as a potential conflict of interest. The handling editor declared a past co-authorship with the authors YZ, YH, SC, MX.
